# On the determination of residual stresses in additively manufactured lattice structures

**DOI:** 10.1107/S1600576720015344

**Published:** 2021-02-01

**Authors:** Tobias Fritsch, Maximilian Sprengel, Alexander Evans, Lena Farahbod-Sternahl, Romeo Saliwan-Neumann, Michael Hofmann, Giovanni Bruno

**Affiliations:** a Bundesanstalt für Materialforschung und -prüfung (BAM), Unter den Eichen 87, 12205 Berlin, Germany; b Siemens Energy GmbH & Co. KG, Huttenstrasse 12, 10553 Berlin, Germany; c Heinz Maier-Leibnitz Zentrum (MLZ), Technische Universität München, 85478 Garching, Germany; d University of Potsdam, Institute of Physics and Astronomy, Karl-Liebknecht-Strasse 24–25, 14476 Potsdam, Germany

**Keywords:** additive manufacturing, laser powder bed fusion, residual stress, principal stress components, neutron diffraction, cellular structures, lattice structures

## Abstract

The determination of residual stresses becomes more complicated with increasing complexity of the structures investigated. Additive manufacturing techniques generally allow the production of ‘lattice structures’ without any additional manufacturing step. These lattice structures consist of thin struts and are thus susceptible to internal stress-induced distortion and even cracks. In most cases, internal stresses remain locked in the structures as residual stress. The determination of the residual stress in lattice structures through nondestructive neutron diffraction is described in this work. It is shown how two difficulties can be overcome: (*a*) the correct alignment of the lattice structures within the neutron beam and (*b*) the correct determination of the residual stress field in a representative part of the structure. The magnitude and the direction of residual stress are discussed. The residual stress in the strut was found to be uniaxial and to follow the orientation of the strut, while the residual stress in the knots was more hydro­static. Additionally, it is shown that strain measurements in at least seven independent directions are necessary for the estimation of the principal stress directions. The measurement directions should be chosen according to the sample geometry and an informed choice on the possible strain field. If the most prominent direction is not measured, the error in the calculated stress magnitude increases considerably.

## Abstract 2

A procedure is outlined for the neutron diffraction determination of residual stress in additively manufactured lattice structures.

## 1. Introduction   

Additive manufacturing (AM) technologies promise dramatic advances in many industrial aspects including part design, production flexibility, and reductions of time to market and scrap. However, several challenges still need to be addressed, especially those linked to the materials science aspects of AM. In particular, for the laser powder bed fusion (L-PBF) AM technique, microstructures are often unconventional and residual stress (RS) is always present after production (Serrano-Munoz *et al.*, 2020 ▸; Mishurova *et al.*, 2019 ▸; Thiede *et al.*, 2018 ▸; Wang *et al.*, 2017 ▸).

For the evaluation of the structural integrity of the material, a nondestructive estimation of RS is essential (Hutchings *et al.*, 2019 ▸). Today the characterization of RS in AM parts is mainly performed by destructive or semi-destructive techniques [*e.g.* the contour method (Ahmad *et al.*, 2018 ▸; Moat *et al.*, 2011 ▸; Vrancken *et al.*, 2014 ▸), hole drilling (Casavola *et al.*, 2009 ▸) and bridge curvature measurements (Kruth *et al.*, 2010 ▸; Bagg *et al.*, 2016 ▸; Mishurova *et al.*, 2017 ▸)]. Many examples of nondestructive RS investigations are focused on surface investigations by means of laboratory X-ray diffraction (XRD) (Mercelis & Kruth, 2006 ▸; Vrancken *et al.*, 2013 ▸). For the determination of bulk RS, laboratory XRD needs incremental layer removal (Evans *et al.*, 2005 ▸). This technique requires extensive sample preparation and is also time consuming. However, the use of large-scale facilities (*i.e.* synchrotrons and neutron sources) is receiving increased attention, since synchrotron radiation and neutron diffraction allow nondestructive determination of RS distributions in the bulk of AM materials. The only nondestructive technique suitable for truly triaxial bulk RS characterization with a millimetre spatial resolution is neutron diffraction (ND) (Boin & Wimpory, 2016 ▸). Furthermore, Moat *et al.* (2011 ▸) compared ND and the contour method and showed that nondestructive and destructive measurements are in good agreement. It is to be noted that the spatial resolution of the contour method is superior to that of ND, but the contour method can only detect unidirectional displacement and, therefore, is best adapted to uniaxial stress states.

Wang *et al.* (2016 ▸, 2017 ▸, 2018 ▸) reported a significant body of research using ND on Inconel 625 (IN 625). They showed that AM IN 625 exhibits a higher stress relaxation rate under operating conditions and lower peak and plateau stress than conventionally processed IN 625. They attributed these differences to different textures and grain sizes in the two materials (Wang *et al.*, 2018 ▸).

L-PBF as an AM method enables the production of geometrically complex structures (Hussein *et al.*, 2013 ▸). The high penetration of neutrons makes ND the only tool able to nondestructively investigate the 3D RS state of such complex AM structures (Watkins *et al.*, 2013 ▸). Cakmak *et al.* (2016 ▸) started to use ND to investigate more complex shapes than cuboids. However, the principal stress directions were determined for forged parts (Robinson *et al.*, 2011 ▸), rolled parts (Coules *et al.*, 2017 ▸) and simple cuboids for L-PBF materials (Gloaguen *et al.*, 2020 ▸; Bayerlein *et al.*, 2018 ▸). In the last case, a deviation between the principal stress directions and the geometrical directions was observed. In the literature, there is still a lot of debate on whether the principal directions of stress follow the hatching directions or rather the geometry of the part. This is discussed in detail by Vrancken *et al.* (2013 ▸), who found that the principal stress directions at the surface coincide with the hatching direction of the last layer for all samples.

A significant challenge for ND is the choice of an appropriate stress-free reference *d* spacing (*d*_0_). Wang *et al.* (2017 ▸) and Sochalski-Kolbus *et al.* (2015 ▸) discussed the variability of the *d*_0_ value along the sample height in relation to the microstructure evolution. Also, Wang *et al.* (2017 ▸) recommended the use of a position-dependent *d*_0_, whereas Colegrove *et al.* (2014 ▸) used the average value of three components. A heat-treated *d*_0_ coupon (made of IN 625; Wang *et al.*, 2017 ▸) showed a change in chemical composition compared with the as-built *d*_0_ coupon, leading to a lattice spacing shift. This calls for spatially resolved *d*_0_ measurements for components with at least one large dimension (typically build height).

A further important point in the diffraction stress analysis is the choice of the appropriate gauge volume. It was shown that the size of the gauge volume influenced the RS determined by ND (Bruno *et al.*, 2006 ▸). In general, a larger gauge volume with respect to the sample size will lead to lower RS values as more grains are averaged (Bruno *et al.*, 2006 ▸).

Among complex structures, lattice structures are intended as a replacement of bulk material, so their mechanical properties are often discussed in the literature (Leary *et al.*, 2018 ▸; Farahbod-Sternahl *et al.*, 2019 ▸). However, the presence of RS in such thin-walled structures may lead to significant distortions and cracking of the part (Yadroitsev & Yadroitsava, 2015 ▸; Mukherjee *et al.*, 2017 ▸; Hussein *et al.*, 2013 ▸; Kruth *et al.*, 2012 ▸). Therefore, RS has a significant influence on the mechanical properties but is often left as an unknown factor in the design of the parts. To the best of the authors’ knowledge, no experimental investigations of RS in lattice structures have been reported so far.

Within this work we aim to provide a reproducible methodology and a reliable guideline for the determination of RS in structures with complex lattice geometries (in the following simply ‘lattice structures’), with a focus on the evaluation of the principal stress directions and magnitudes.

## 2. Sample and experiment   

**Figure 1:**
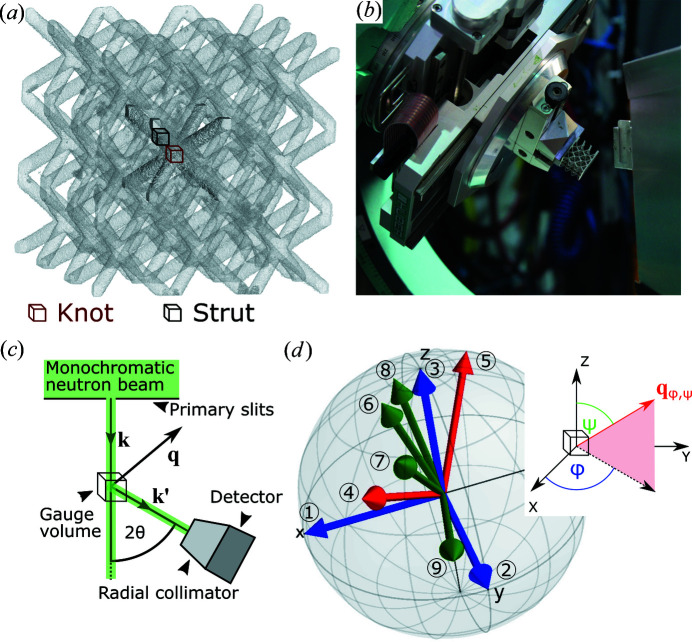
(*a*) 3D-rendered lattice structure (from XCT data) shown with 70% transparency to highlight the central unit cell. Neutron diffraction gauge volumes are indicated in the knot (red) and the strut (black) positions. (*b*) The experimental setup at Stress-Spec and the mounted lattice structure. (*c*) The schematics of the neutron diffraction experiment with primary slits and a radial collimator in front of the detector. (*d*) The nine investigated scattering vectors 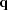: three directions corresponding to the geometrical sample directions (blue, ‘1’ to ‘3’), two directions corresponding to the axial and radial directions of the strut (red, ‘4’ and ‘5’), and four random directions (green, ‘6’ to ‘9’). Each scattering vector is presented in the sample coordinate system and is defined by the azimuthal angle 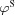 and the polar angle 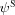 shown in the inset.

The investigation was focused on a lattice structure consisting of 3 × 3 × 3 body centred cubic (b.c.c.) unit cells (UCs). The structure had a strut diameter of 1 mm and a strut length of 10 mm, which reflects the length of the UC room diagonal [see Fig. 1 ▸(*a*)]. The edge length of a UC was therefore 5.77 mm. The sample was produced by Siemens AG, Gas and Power, Berlin, Germany. An EOS M290 printer was used along with a set of Siemens proprietary parameters (the layer thickness was 20 µm).

The material used for production of the lattice structures was IN 625. The nominal chemical composition of the IN 625 is shown in Table 1 ▸.

The ND experiment was performed at the Stress-Spec beamline at the Neutron Reactor FRM II in Garching, Germany (Hofmann *et al.*, 2006 ▸). Fig. 1 ▸(*b*) shows a photograph of the lattice structure mounted on the Euler cradle of the diffractometer. Fig. 1 ▸(*c*) shows a sketch of the Stress-Spec beamline. The gauge volume was defined by a primary slit of 1 × 1 mm and an oscillating radial collimator with a full width at half-maximum equal to 0.5 mm in front of the detector [see Fig. 1 ▸(*c*)]. Such a gauge volume was needed because of the small strut diameter (1 mm). A smaller gauge volume would have led to a statistically insufficient scattered intensity. A larger gauge volume would have exceeded the strut dimensions.

The wavelength of the thermal neutron beam was tuned to 0.142 nm using an Si monochromator. In this way, the diffraction signal of the {311} lattice plane was evaluated around a detector position of 2θ = 84°. The reflection 311 was chosen as it best represents the macroscopic mechanical behaviour of nickel (Holden *et al.*, 1997 ▸). The diffraction elastic constants *E*_311_ = 193.5 GPa and *ν*_311_ = 0.305 were used for the stress calculation (Wang *et al.*, 2016 ▸). These values have been experimentally estimated on IN 625 manufactured by laser metal deposition (Wang *et al.*, 2016 ▸).

The stress-free reference *d* spacing *d*_0_ was chosen as the average of all measured *d* spacings. This assumption is justified by the large number of measurements and independent directions. The problems occurring during the determination of RS in AM materials (with regard to the diffraction elastic constants and to *d*_0_) are discussed by Mishurova *et al.* (2020 ▸).

The full procedure of the calculation of both principal stress and magnitudes is described in Appendix *A* [App appa].

Two positions in the specimen were measured: one gauge volume was placed at the centre of the lattice structure (knot), and another gauge volume was placed in the strut at 2 mm distance to the knot [see Fig. 1 ▸(*a*)]. Nine strain components were determined for each of the two gauge volumes [see Fig. 1 ▸(*d*)]. The counting time was 30 min for each component.

To ensure precise sample mounting and alignment, an Eulerian cradle (χ rotation) was mounted on top of the rotation stage (ω rotation). A second rotation stage (φ rotation) was mounted on the Eulerian cradle together with an additional *x*, *y* and *z* translation table [see Fig. 1 ▸(*b*)].

The alignment of the lattice structure in the neutron beam started with the optical alignment of a knot of an outer UC by means of a theodolite. Afterwards, neutron entrance scans in the **x**, **y** and **z** directions were performed to find the maximum of the diffracted signal on the 2D detector. The maxima of diffraction intensities indicate that the strut was approximately the size of the gauge volume but slightly smaller. A plateau would be expected if the strut was significantly smaller than the gauge volume.

The movement along the strut (*i.e.* along the room diagonal of the b.c.c. cell structure) was performed by using the same displacement along all three translation axes. This means that, during the ND experiment, the strut was assumed to be a perfect room diagonal. This assumption was verified by means of a nominal–actual comparison between an X-ray computed tomography (XCT) scan and the computer aided design (CAD) file (see Fig. 8 in Appendix *B* [App appb]). Using the data elaboration and visualization software *VGSTUDIO MAX 3.2* (https://www.volumegraphics.com/de/produkte/vgstudio-max.html), a plane was fitted to the compression plate [at the top of the lattice structure; see Fig. 1 ▸(*b*)] and a cylinder to the strut that was investigated. The angle between the plane and the cylinder axis was found to be 35.21°. The deviation from the nominal angle of 35.27° is below 0.3%, thereby showing that the angle of the strut was undistorted (on average). Local distortions could also influence the ND experiment, but a distortion between the printed strut and the nominal cylinder is not perceptible within the resolution of the XCT scan (around 50 µm). The difference between the actual and the nominal strut shape is dominated by the roughness of the strut. The roughness *P*_a_ was determined (using XCT data) to be around 40 µm [see Fig. 8[App appb](*b*)]. With respect to the strut diameter (1 mm) the roughness is considered to not be critical. An anomalously large roughness would lead the gauge volume to probe empty regions.

We can conclude that the positioning precision of the gauge volume in the strut was better than 50 µm and similar to the surface roughness. This result goes beyond the typical precision achievable in ND measurements (Standard ISO 21432:2019: *Non-destructive testing: standard test method for determining residual stresses by neutron diffraction*).

Additionally, possible errors in the alignment were corrected by ‘mirror’ measurements for the two components ‘1’ and ‘2’ [see Fig. 1 ▸(*d*)]: each of them was measured at φ and φ +180°. A possible pseudo-strain was averaged out by this method (Holden *et al.*, 2015 ▸). The average peak position was used for strain and stress analysis. Remarkably, no significant differences were found between the two measurements [see Fig. 8[App appb](*c*)]. This confirmed that the alignment was at its optimum.

Another source of error in the interpretation of RS results would be a locally variable microstructure. Therefore, a sister sample (same build job, but different position on the build plate) of the lattice structure was cut along the strut by means of electric wire erosion. The cut face was diamond polished step-wise down to 1 µm and finished by colloidal silica. A Gemini 1530VP (Leo/Zeiss) scanning electron microscope was used together with the detector e-flash HR+ and the software *Esprit1.9* (both Bruker) for electron backscatter diffraction (EBSD) investigations. The sample chamber was evacuated to 5 Pa to prevent electrical charging and electrical drifting of the sample.

**Figure 2:**
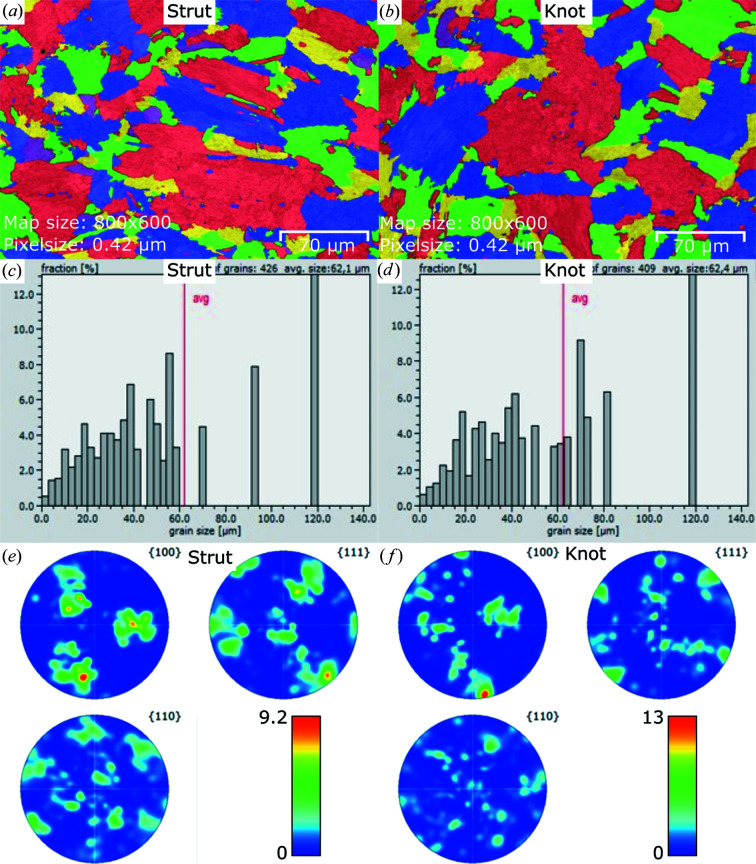
EBSD images: (*a*) at the strut position and (*b*) at the knot position. The colours just indicate different grains and are not linked to their orientation. The grain size distribution [calculated from (*a*) and (*b*)] of the single unit cell at (*c*) the strut and at (*d*) the knot. Pole figures measured at (*e*) the strut position and (*f*) the knot position.

Fig. 2 ▸ shows the EBSD images, the grain size distribution and the texture (pole figures) at the strut and knot positions. Neither the grain size nor the texture shows significant differences between the strut and the knot positions.

## 3. Results and discussion   

The principal stress directions were calculated using a Python script. The six independent stress components were extracted from the nine strain measurements, and then the eigenvalues and eigenvectors of the stress tensor were calculated. The procedure is outlined in Appendix *A* [App appa] (see also Hauk, 1997 ▸; Noyan & Cohen, 2013 ▸; Hutchings & Krawitz, 1992 ▸; Spieß *et al.*, 2015 ▸).

**Figure 3:**
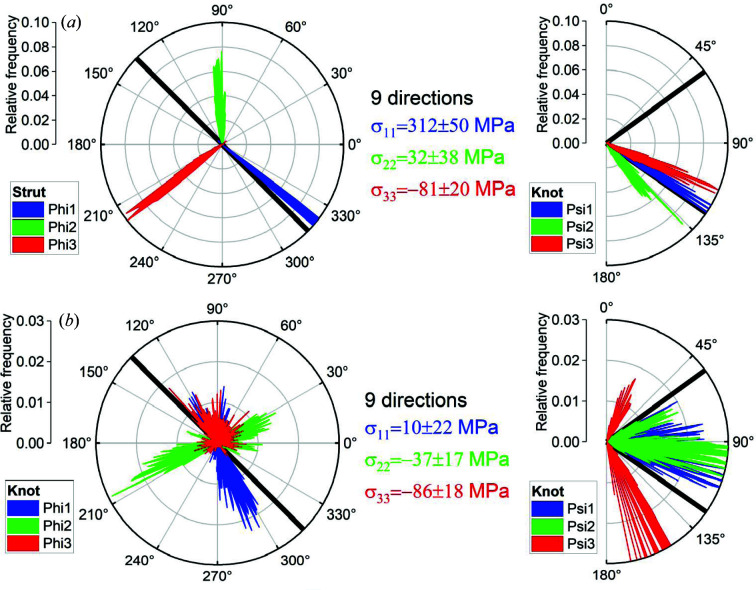
The results of the principal direction estimation for (*a*) the strut and (*b*) the knot in the sample coordinate system in terms of the eigenvalue σ_*ii*_ (middle), the azimuthal angle 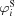 (left) and the polar angle 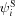 (right) of the corresponding eigenvector (blue, green, red).

All results represent the statistical evaluation of 1000 calculations to estimate an error band for all principal stress directions and magnitudes. Each calculation used random strain values within the experimental strain error as input for the stress calculation. Figs. 3 ▸(*a*) and 3 ▸(*b*) show the results of the principal direction calculations using all nine measured directions [see Fig. 1 ▸(*d*)] for, respectively, the strut and the knot of the lattice structure, depicted as cubes in Fig. 1 ▸(*a*).

The resulting eigenvectors are presented through their azimuthal and polar angles 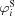 and 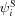 in the sample coordinate system 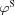 (left) and 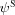 (right). The definitions of 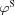 and 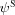 are also sketched in the inset of Fig. 1 ▸(*d*). The corresponding eigenvalues are given in the form of the stress values σ_*ii*_.

Fig. 3 ▸(*a*) depicts the orientation of the three principal directions (blue, green and red) for the gauge volume positioned in the strut. Sharp peaks were observed for all three principal directions. The stress values reported in the figure represent the eigenstress along the respectively coloured eigenvectors. The strut [Fig. 3 ▸(*a*)] shows a maximum principal stress of σ_11_ = 312 ± 50 MPa along 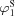 = 310° and 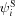 = 122°. This direction corresponds to the strut orientation in the sample coordinate system 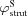 = 315° and 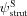 = 125.3°. The geometrical orientation of the strut is marked with thick black lines.

The second principal stress component (green) showed a value smaller than its error. This is therefore understood as zero. A small compressive stress value (−80 MPa) is observed for the third stress component (red), which corresponds to a radial component with respect to the strut.

Such results indicate a nearly uniaxial stress state, as it would be expected for an elongated cylindrical shape. With the gauge volume having approximately the same size as the strut diameter, the macroscopic stress is averaged out for the radial and hoop stress components (green and red) in the strut. On the one hand, ND is the only suitable tool to probe the stress state in such filigree structures; on the other hand, the size of the gauge volume cannot be made smaller (to probe possible stress profiles in the struts). We therefore recommend our method as the best compromise for a reliable evaluation of the 3D stress state in lattice structures.

The same calculations as for the strut were performed for the knot in the centre of the lattice structure. The results are presented in Fig. 3 ▸(*b*) and in Figs. S1(*b*), S3 and S4 in the supporting information. In comparison with the results for the strut [Fig. 3 ▸(*a*)], the stress magnitude σ_11_ is small, while σ_22_ and σ_33_ are the same within the error bars. The stress state is basically hydro­static. In fact, the distributions of 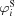 and 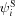 are nearly isotropic. No prominent direction can be identified. That correlates well with the fact that the knot is positioned at the junction of four struts. While it is expected that the RS state is more hydro­static at the knot than at the strut, it is not intuitive that the principal stress directions are dominated by the part geometry rather than by the scan strategy. Indeed, although Vranken *et al.* (2013 ▸) and Bayerlein *et al.* (2018 ▸) reported that the scan strategy dominates, Thiede *et al.* (2018 ▸) showed that the principal stress directions coincide with the geometrical ones.

Another reason for the significant difference between the stress states in the strut and at the knot position could be a difference in the microstructure. However, no difference in grain size or grain misorientations was found (see Fig. 2 ▸ for details).

A comparison between Figs. 3 ▸(*a*) and 3 ▸(*b*) shows that the knot possesses significantly lower stress values and a broader distribution for the principal stress direction. [Note that for the sake of clarity Fig. 3 ▸(*a*) has a different scale for the relative frequency compared with Fig. 3 ▸(*b*).]

By reducing the number of measured directions from nine to seven for the calculation of eigenvalues and eigenvectors, the principal directions do not change significantly. The only difference is that the algorithm returns the exact opposite unit eigenvectors (see the supporting information Figs. S1 and S2). Interestingly, the results of the calculation of the principal stress directions are independent of the choice of the seven directions (see Fig. S2), but the principal stress magnitudes do depend on this choice. The cause for the dependence is yet unknown and this investigation is left for future work. We can, therefore, conclude that the use of seven measurement directions cannot be recommended. Note also that this result suggests an alternative strategy: one could measure seven directions to determine the principal stress directions and then measure the principal strains along such directions. This would, however, bring the total number of measurements to ten.

**Figure 4:**
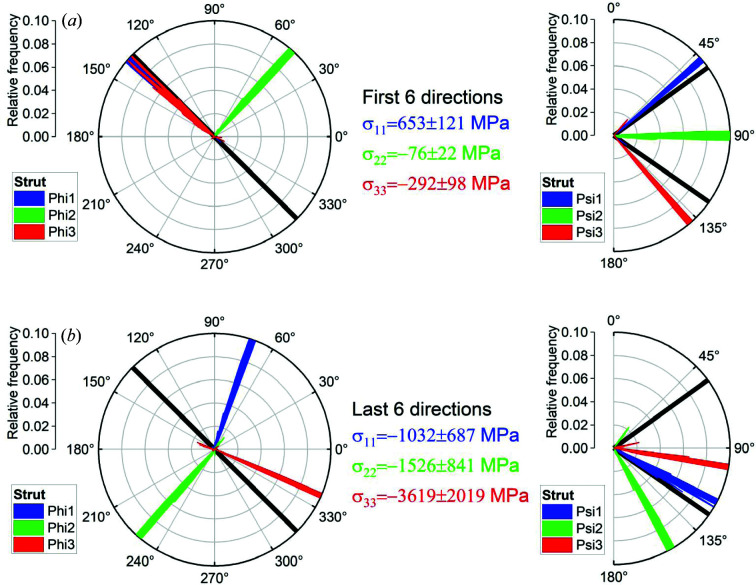
The results of the principal direction estimation for the strut position for the directions (*a*) ‘1’–‘6’ and (*b*) ‘4’–‘9’ in the sample coordinate system in terms of the eigenvalue σ_*ii*_ (middle), the azimuthal angle 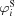 (left) and the polar angle 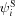 (right) of the corresponding eigenvector (blue, green, red).

The reduction of the data sets to six measured directions has a major impact on the results [exemplified in Fig. 4 ▸(*a*) for the strut]. Both the principal stress values and the principal directions are significantly different from those obtained using nine [Fig. 3 ▸(*a*)], eight (Fig. S1) and seven directions (Fig. S2). Unrealistically, the calculated principal directions are uncorrelated with the strut orientation. Moreover, the choice of the six directions has a large impact on the principal stress directions [compare Figs. 4 ▸(*a*) and 4 ▸(*b*)] and on the principal stress magnitudes [they become higher than the yield strength of IN 625 bulk material; 400 MPa (Yangfan *et al.*, 2019 ▸) to 793 MPa (Gao & Zhou, 2018 ▸)]. This leads to the conclusion that selecting six components would not yield meaningful strain values to correctly calculate the principal directions in lattice structures. This fact goes beyond any continuum mechanics textbook, where it is stated that the stress and strain tensors are unambiguously determined by six independent measurements. It is apparent that residual stress determination in AM lattice structures requires a paradigm shift.

An artificial increase of the measurement error by a factor of 5 (from about 100 to 500 µstrain) for each direction led to a broader angular distribution of 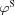 and 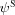 along with an increased error on the stress magnitude (by a factor of 3, *i.e.* compare Fig. 3 ▸ with Fig. S5). While for the strut the principal directions are still recognizable with large measurement errors of about 500 µstrain [see Fig. S5(*a*)], the directions for the knot [see Fig. S5(*b*] are lost. This indicates that an increase of the measurement error would yield totally unreliable results of the principal directions, especially if strain values are low (*i.e.* in the knot). We can conclude that for the determination of stresses in complex geometries it is extremely important that the measurement error be kept as low as possible (at the cost of beamtime, for instance).

**Figure 5:**
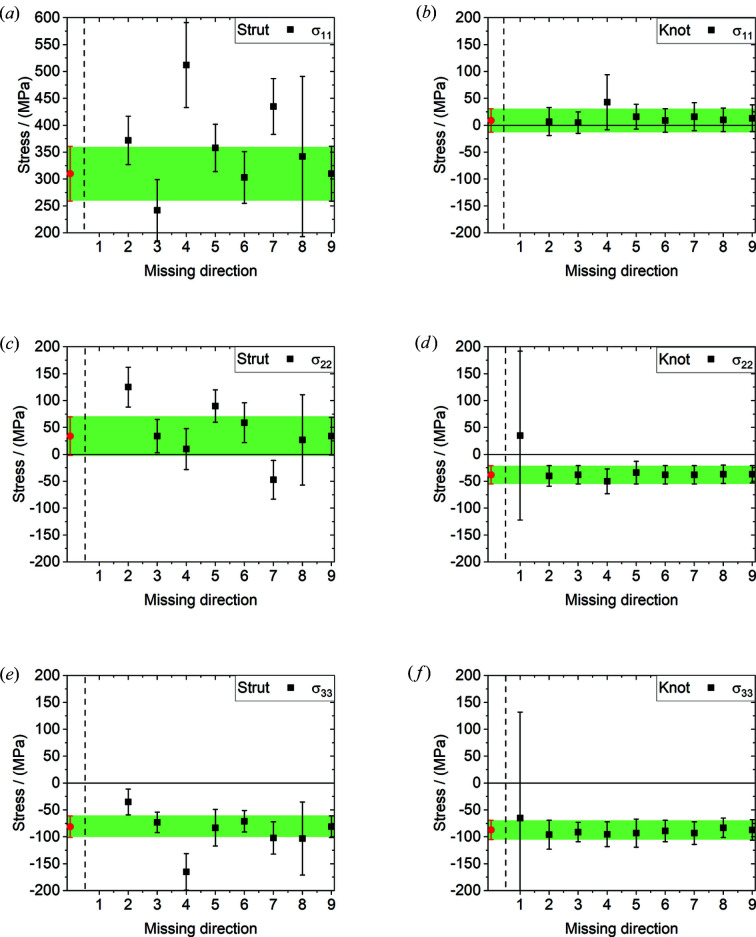
The principal stress magnitudes (σ_11_, σ_22_, σ_33_) are shown for the use of eight measured directions for stress calculation for (*a*), (*c*), (*e*) the strut and (*b*), (*d*), (*f*) the knot positions. The missing direction on the *x* axis refers to the direction number in Fig. 1 ▸(*d*). The red data points represent the calculation with all nine measured components. The green band represents the error bar for the calculation with nine strain measurements.

The principal stress magnitudes are shown in Fig. 5 ▸ for the case of nine measured directions (red points) and for the nine possible combinations with eight measured directions for both the strut [Figs. 5 ▸(*a*), 5 ▸(*c*) and 5 ▸(*e*)] and the knot [Figs. 5 ▸(*b*), 5 ▸(*d*) and 5 ▸(*f*)]. The red data points represent the principal stress determined with all nine measurements. The green band marks the error range of the stress magnitude calculated using all nine measured directions.

In the case of the strut [see Figs. 5 ▸(*a*), 5 ▸(*c*) and 5 ▸(*e*)] the results for eight directions are not always in agreement with the calculation for nine. In particular, if a significant direction (‘4’ or ‘7’) is missing, the results do not match the calculation for nine directions. Direction ‘4’ is the axial component of the strut and direction ‘7’ reflects the random direction with the least angular deviation from direction ‘4’ [see Fig. 1 ▸(*d*)].

The principal stress magnitudes do not vary with the choice of the missing measured direction in the case of the knot, but they do for the strut. This confirms that the stress state is more hydro­static at the knot position, and lower stress magnitudes are found than for the strut.

## 4. Concluding remarks   

**Figure 6:**
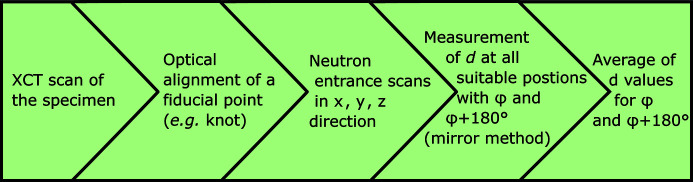
Schematic of the experimental procedure for the determination of residual stress in lattice structures.

**Figure 8:**
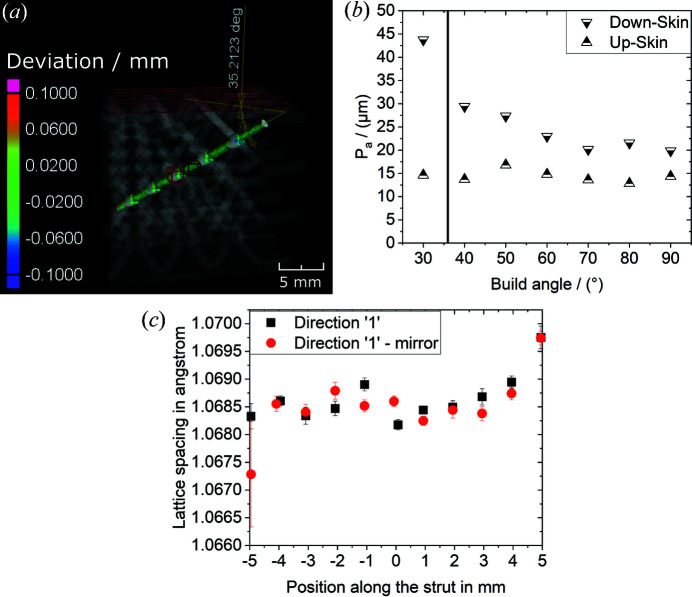
(*a*) A nominal-to-actual comparison of the investigated strut: the experimentally determined room diagonal of the lattice structure has an angle of 35.21° to the upper horizontal plate, while the nominal angle between strut and plate would be 35.27°. In (*a*) the struts are shown as shaded, and the positions of the gauge volumes used in neutron diffraction measurements are also displayed in red for the knot and in black for the strut. (*b*) A roughness analysis on a single strut by means of X-ray computed tomography for various build angles α. The strut of the lattice structure was built with α = 35.27°, indicated by a vertical line. (*c*) The measured lattice spacings for various positions along the strut. The black squares and red circles represent the measurements along direction ‘1’ and the opposite direction (mirror).

We have elaborated a robust procedure to determine residual stress in lattice structures using neutron diffraction (Fig. 6 ▸). We showed that the alignment of the lattice structure in the neutron beam needs prior X-ray computed tomography scans to acquire the exact internal geometry of the part (a classic coordinate measuring machine would be unable to detect the internal structure). We also showed that, because of the filigree structure, mirror scans (*i.e.* entrance scans with the sample at two opposite azimuthal angular positions) during the neutron diffraction experiment are needed to avoid pseudo-strains.

For the calculation of the strain-free reference, we found that the grand average of all measurements in the structure represents a good working hypothesis (it represents the boundary condition of vanishing stress within the whole volume), provided a large number of measurements are undertaken. The same *d*_0_ value was used for the knot and the strut positions, since the microstructure in terms of grain size and texture was found to be similar at the two locations. Moreover, the distance between the two points was only around 2 mm, while previous work (see *Introduction* [Sec sec1]) indicates significant *d*_0_ variation only for much larger distances. A detailed measurement of the chemical variations would need to be considered in the case of larger distances between the measurement points. If the experimental procedure described in the manuscript were to be extended to map the residual stress over the whole lattice structure, the similarity of the microstructure between all struts and knots would need to be proven.

Importantly, we found that measurements in nine directions (at each point) are needed in lattice structures to reliably calculate both the principal stress values and the principal directions. Contrary to present textbooks and to theory, we showed that six independent directions of measurement are not sufficient to find either the principal stress directions or the corresponding stress values in the lattice structures. At least seven directions are required for the calculation of the principal directions of stress; such a strategy would also guarantee that the principal stress directions are insensitive to the choice of the measurement directions. In contrast, the principal stress magnitudes were sensitive to the choice of the directions used for calculation, even using eight measurement directions. These facts demonstrate the peculiarity of complex additively manufactured structures. Lattice structures showcase that the classic testing tools need to be critically evaluated and eventually redefined as much as the design of AM parts. They also show that even some commonly accepted theoretical concepts need to be systematically verified by experiments. In fact, we showed that, if measurements are not carried out with the best possible precision (even at the price of costs and time), erroneous (as opposed to just approximate) results are obtained.

Finally, we showed that the residual stress ellipsoid aligns with the substructure of the lattice: the stress state in the strut is approximately uniaxial, while the stress state in the knot has a strong hydro­static character.

The procedure and insights reported in this work will enable the experimental evaluation of the spatial distribution of residual stress within whole lattice structures, *e.g.* stress maps of the whole lattice structure with the resolution of the strut diameter, and in general complex geometries, thereby allowing validation of theoretical models (*e.g.* finite element method codes). One could imagine *in situ* testing to correlate the residual stress results with the mechanical failure under load.

## 5. Data availability   

The data sets generated and/or analysed during the current study are available from the corresponding author on reasonable request.

## 6. Author contributions   

The conceptualization was done by TF. Sample preparation and measurements were performed by TF, MS, LFS, RSN, MH and AE. TF and MS evaluated and visualized the data. TF, MS, AE and GB discussed the results. TF wrote the main manuscript, and all reviewed the manuscript.

## Supplementary Material

Supporting information file. DOI: 10.1107/S1600576720015344/fs5192sup1.pdf

## Figures and Tables

**Figure 7:**
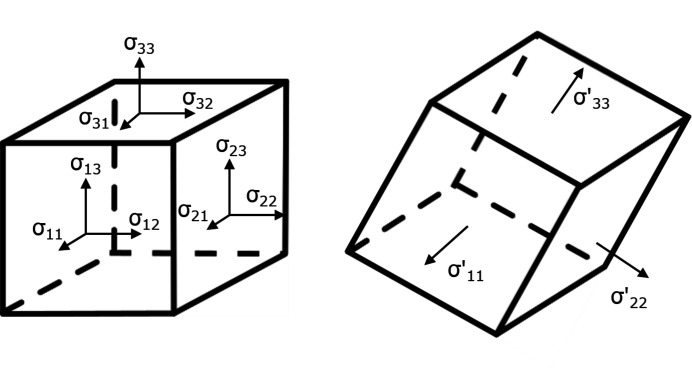
Schematic representation of the eigenvalue decomposition on a unit cube. The unit cube on the right is rotated in such a way that all shear stress components vanish.

**Table 1:** Chemical composition of IN 625 in wt% (https://www.specialmetals.com/assets/smc/documents/inconel_alloy_625.pdf)

Cr	Mo	Fe	Ta + Nb	Mn	Si	Ti	Al	Ni
20–23	8–10	5	3.14–4.15	0.5	0.5	0.4	0.4	Balance

## Appendix A Calculation of principal residual stress directions and magnitudes

We used Bragg’s law to calculate the lattice spacing *d* from the measured diffraction angle 2θ for the {311} lattice plane for a given pair of azimuthal angle  and polar angle  in the sample coordinate system [see Fig. 1 ▸(*d*)]. We then used the equation for diffraction-based stress analysis (Hauk, 1997 ▸; Spieß *et al.*, 2015 ▸; Noyan & Cohen, 2013 ▸; Hutchings & Krawitz, 1992 ▸):

The lattice plane {311} was not indicated in the strain ε, the lattice spacings *d* and *d*_0_, the Poisson ratio ν, or the elastic modulus *E* in equation (1) for the sake of brevity. Also, *n* ∈ [1, 9] is the index of the measured direction. As we measured nine directions, we obtained an overdetermined linear equation system and solved it by a least-squares method for the six unknown stress components . The symmetry of the stress tensor () was used to calculate the full tensor. An eigenvalue decomposition of the stress tensor (visualized in Fig. 7 ▸) was carried out using equation (2):

It resulted in the eigenvectors **v**_1_, **v**_2_, **v**_3_ representing the principal stress directions, and in the eigenvalues  representing the principal stress magnitudes.

The procedure was implemented in a Python script.

## Appendix B Sample alignment in neutron diffraction measurements

We performed an XCT scan on the lattice structure that was investigated by ND to prove that the investigated strut is straight and has the correct angle towards the plate. The XCT scan was performed on a GE v|tome|x 180/300L using a 300 kV reflection target. A voltage of 250 kV and a current of 100 µA were applied and the total scan time was 180 min. The voxel size was 25 µm. *VG Studio MAX 3.2* was used for the nominal-to-actual comparison between the XCT scan and the CAD file [see Fig. 8 ▸(*a*)].

A single strut was scanned with higher magnification on the same system, but using the 180 kV transmission target. For this experiment, the X-ray source was operated at 125 kV and 60 µA and the total scan time was 70 min. A voxel size of 2.1 µm was achieved. This allowed the extraction of a line profile at the Up-skin and the Down-skin for roughness analysis. This roughness is plotted in Fig. 8 ▸(*b*) as a function of the build angle of the single struts. The vertical line indicates the build angle of a strut implemented in the investigated lattice structure.

Fig. 8 ▸(*c*) depicts the lattice spacing of a strut obtained by ND. We measured along the full strut (length = 10 mm) twice. While the first measurement was performed using (φ, ), the second scan was performed using (φ + 180°, ).
