# Supplementary material for: On the determination of residual stresses in additively manufactured lattice structures
Source: J Appl Crystallogr. 2021 Feb 1;54(Pt 1):228–36. doi: 10.1107/S1600576720015344 (PMC7941307; doi:10.1107/S1600576720015344)
Supplement: Supplementary file 1 [file j-54-00228-sup1.pdf]

JOURNAL OF APPLIED CRYSTALLOGRAPHY

Volume 54 (2021)

Supporting information for article:

On the Determination of Residual Stresses in Additive Manufacturing Lattice Structures

Tobias Fritsch, Maximilian Sprengel, Alexander Evans, Lena Farahbod-Sternahl, Romeo Saliwan-Neumann, Michael Hofmann and Giovanni Bruno

Evaluation of the principal stress values and directions using 8, 7, and 6 independent strain values.

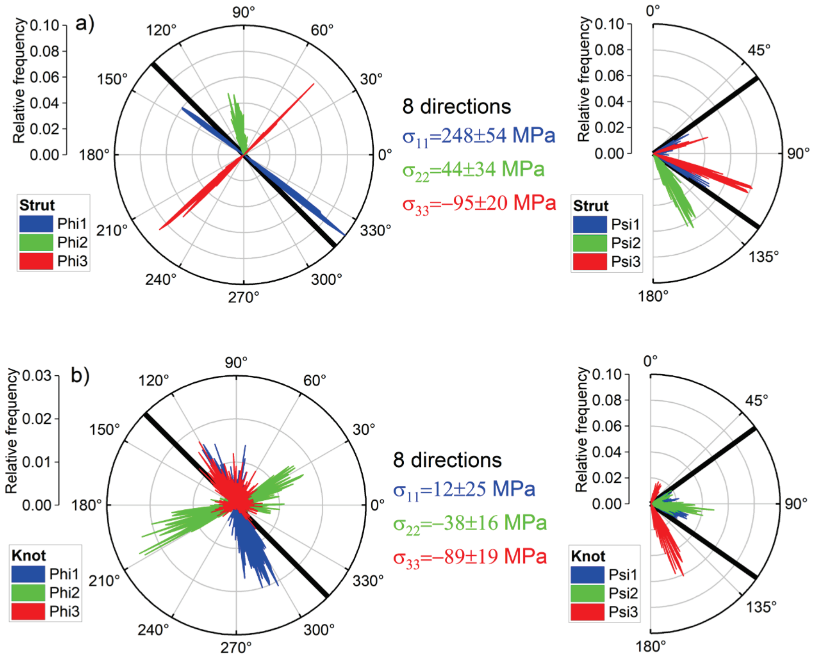

Figure S1: The results of the principal direction estimation for a) the strut and b) the knot in the central unit cell (UC) of the lattice structure in the sample coordinate system: the eigenvalues σii (middle) as well as the azimuthal angle ϕ_i^S (left) and the polar angle ψ_i^S (right) of the corresponding eigenvector (blue, green, red) are shown. Each color bar represents a principal stress component. These results are presented for directions '1-8' (see Figure 1d)

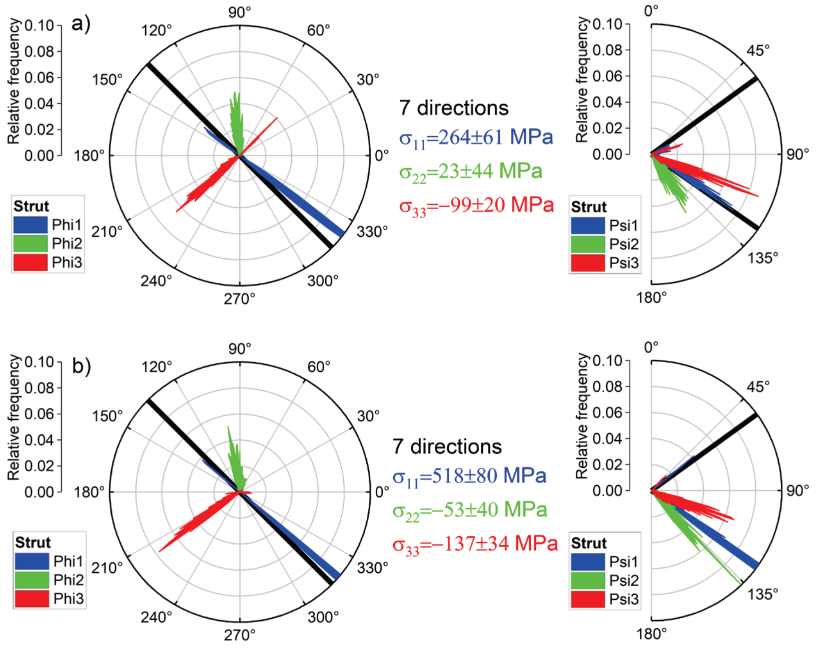

Figure S2: The results of the principal direction estimation for the strut in the central UC of the lattice structure in the sample coordinate system: the eigenvalues σii (middle) as well as the azimuthal ϕ_i^S (left) and polar ψ_i^S (right) angles of the corresponding eigenvector are shown. Each color bar represents a principal stress component. These results are presented for a) directions '1-7' (see Figure 1d) and b) the seven directions with the lowest µstrain value ('1-3, 5-6, 8-9').

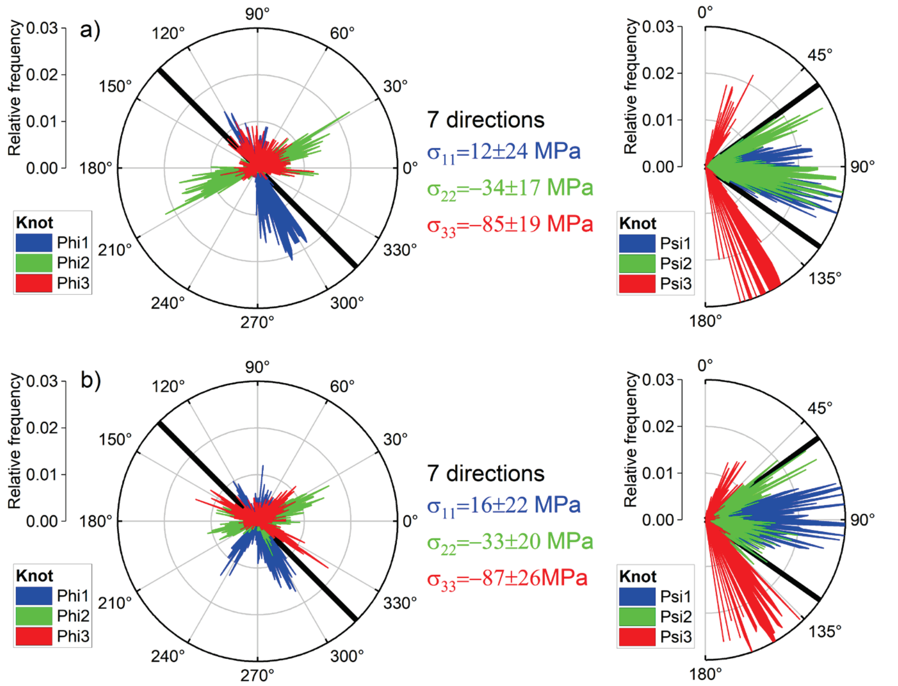

Figure S3: The results of the principal direction estimation for the knot in the central UC of the lattice structure in the sample coordinate system: the eigenvalues σ_{ii} (middle) as well as the azimuthal ϕ_i^S (left) and polar ψ_i^S (right) angles of the corresponding eigenvector are shown. Each color bar represents a principal stress component. These results are presented for a) directions ‘1-7’ (see Figure 1d) and b) the seven directions with the lowest µstrain value (‘1-3, 5-6, 8-9’).

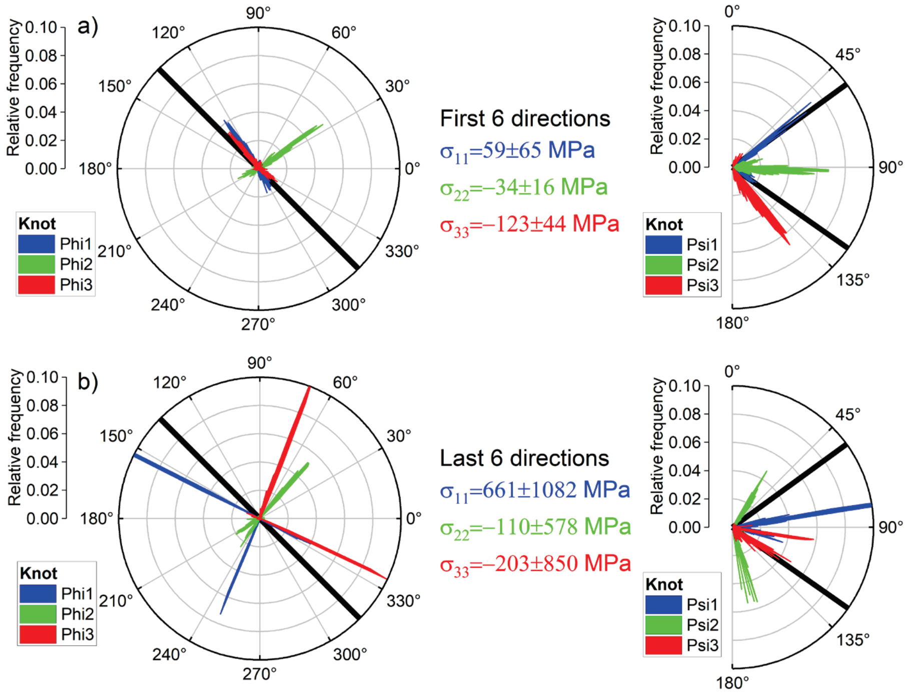

Figure S4: The results of the principal direction calculation for the knot position using only six measurements: using the directions a) ‘1-6’ and b) ‘4-9’ (see the sample coordinate system in Figure 1d). The eigenvalues σ_{ii} (middle), the azimuthal angle ϕ_i^S (left), and the polar angle ψ_i^S (right) of the corresponding eigenvector (blue, green, red) are shown.

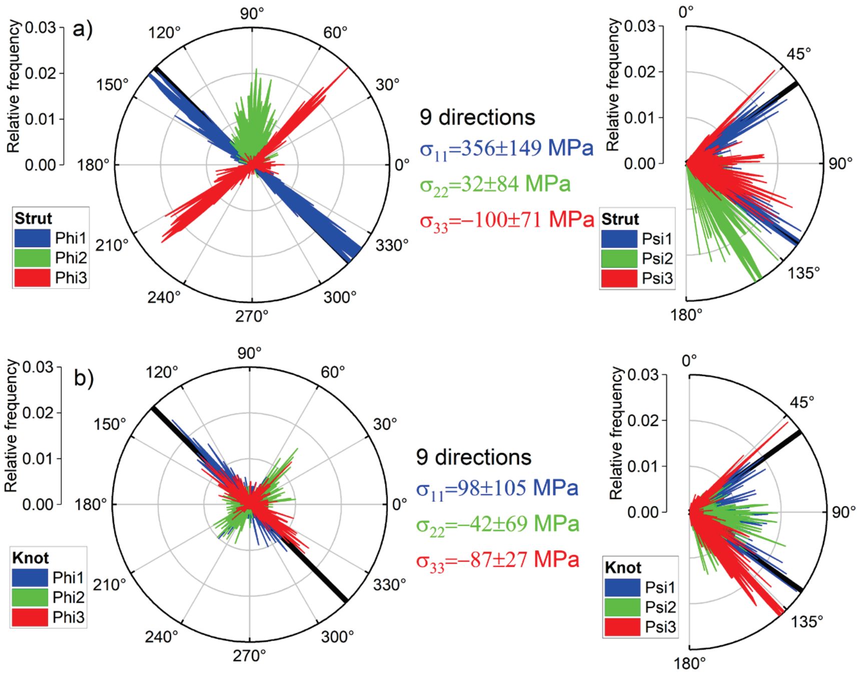

Figure S5: The calculated principal direction for a) the strut and b) the knot in the sample coordinate system: the eigenvalues σ_{ii} (middle), the azimuthal angle ϕ_i^S (left), and the polar angle ψ_i^S (right) of the corresponding eigenvector (blue, green, red) under the assumption of an artificial error of 500 µstrain on every measured strain value. This is to compare with Figure 2a.
